# The Practitioners' Bookshelf

**Published:** 1893-01-21

**Authors:** 


### THE PRACTITIONERS' BOOKSHELF.

### THE BEGINNINGS OF LIFE.*

It is a great advantage to the beginner in biology to have a text-book written by a medical man—always supposing, of course, that the medical man can really write. Dr. Campbell belongs to the class of book constructors who take the trouble to understand their business. Biology, the science of living things, is one of those subjects which everybody either wants or ought to want to know something about, and the kind of knowledge wanted is not mere knowledge of matters of fact. The general reader, and thinker, wants indeed to know the facts; but he wants to know them in relation to other facts—to understand them, in short. He does not wish to see things out of proportion, to take a biological mouse for an elephant, or *vice versa*. Moreover, he likes to see the facts in a historical setting. He does not wish to announce to his friend, in conversation, a fact as a new discovery which is two or three centuries old. But all these mistakes are constantly being made by even intelligent people who come new to the subject. Medical students themselves, to say nothing of practitioners, are frequent blunderers in this regard, and in them such blundering is inexcusable.

Life, as it manifests itself in visible and tangible forms, is the great business of the scientist, the student of medicine, and the medical practitioner. The very first necessity of successful work among living forms is that something should be known about them. The medical student and the practitioner deal with living forms of the most highly specialised variety, of the greatest physiological and absolute value. The first thing, then, that all people who venture to touch the subject of medicine should do is to acquire a clear and intelligent knowledge of living forms; to find out all about their various modes of life, nutrition, development, specialisation, and death. Every medical student, indeed, should become a biologist at the beginning of his career, and every medical practitioner should continue to be an intelligetn biologist throughout his life.

But if this be what is naturally demanded of medical men, it is essential that biology shall be reduced to its simplest elements, and shall be so presented by teachers to students that there shall be no unnecessary difficulty in understanding it. This task of making biological science simple, easy of comprehension, and interesting is what Dr. Campbell has set himself; and he has succeeded excellently well. The book, however, though simple, is thoroughly scientific; and is only popular in the sense that Dr. Campbell has put himself in the place of the reader, and has done what every teacher is morally bound to do—made "the crooked straight and the rough places plain." But it is not by medical students alone that such a book as this should be read and studied; it is the very book for the high school and the college. One would think that the clergy, too, who, as public teachers, ought to be familiar with the scientific lines on which men's thoughts are more and more running every day, would be glad of a book of the kind. It is small—containing less than 300 quarto pages—and cannot be very expensive. Moreover, it is thoroughly trustworthy and up to date; the work of a competent teacher and honest scientific worker. We regret that, from want of space, our notice is necessarily of a cursory character; but the merits of the little book are great, and the service it can render to intelligent readers will be found to be also great.

### THE HYGIENE OF THE EAR.†

Dr. Cozzolino's little work has already run through four editions, and has been translated into six European languages, surely good evidence that it has proved of value to the medical profession and the public. The hygiene of the ear in infancy, childhood, adult life is dealt with in a plain and impressive manner, and the concluding chapter on general contains many valuable suggestions.

* "Elementary Biology." By H. J. CAMPBELL, M.D. Lond., Demonstrator of Biology and Physiology at Guy's Hospital. (London, Swan Sonnenschein and Co.; New York, Macmillan and Co.)

† "The Hygiene of the Ear." By Dr. VINCENZO COZZOLINO (Naples). Translated from the Fifth Italian Edition by JAMES ERSKINE, M.A., M.B., Glasgow. Pp.52 8vo. Baillière, Tindall, and Cox, London, 1892.

